# Musculoskeletal health and osteoarthritis in the UK army – the ADVANCE study (Armed Services Trauma Rehabilitation Outcome Study)

**DOI:** 10.1186/1471-2474-16-S1-S13

**Published:** 2015-12-01

**Authors:** Alexander Bennett

**Affiliations:** 1 Rheumatology and Rehabilitation, DMRC Headley Court, Epsom, Surrey KT18 6JW, UK

## 

Trauma is a well recognised risk factor for the future development of osteoarthritis (OA). The Armed Services Trauma and Rehabilitation outcome study (The ADVANCE study) is a 20 year cohort study comparing medical and psychosocial outcomes of military personnel both exposed and not exposed, to significant trauma.

The Bio-Mil-OA study, a sub-study of the ADVANCE study, is an ideal opportunity to investigate the predictive value of biomarkers in joint pain and osteoarthritis.

The ADVANCE study is a 20 year cohort study of 600 combat casualties and 600 matched non exposed participants investigating the predictive value of biomarkers and trauma on the long term development of pain and OA in the hip and knee. Validated serum biomarkers for OA, knee and hip radiographs and patient reported outcomes for pain and function will be taken at baseline and at 5 years.

The predictive value of the biomarkers in predicting OA development and progression and joint pain in those exposed to different levels of trauma will be investigated using quantitative immunoassays for catabolic markers of cartilage matrix degradation.

